# Determinants promoting and hindering physical activity in primary school children in Germany: a qualitative study with students, teachers and parents

**DOI:** 10.3389/fpubh.2024.1280893

**Published:** 2024-02-02

**Authors:** Louisa Sell, Berit Brandes, Mirko Brandes, Hajo Zeeb, Heide Busse

**Affiliations:** ^1^Department of Prevention and Evaluation, Leibniz Institute for Prevention Research and Epidemiology, BIPS GmbH, Bremen, Germany; ^2^Institute for Public Health and Nursing- IPP, Bremen University, Bremen, Germany; ^3^Department of Epidemiological Methods and Etiological Research, Leibniz Institute for Prevention Research and Epidemiology, BIPS GmbH, Bremen, Germany; ^4^Health Sciences Bremen, University of Bremen, Bremen, Germany

**Keywords:** physical activity, perspectives of children, primary schools, teachers, parents, socio-ecological model, qualitative research, focus groups

## Abstract

**Background:**

Determinants affecting children’s physical activity (PA) at an early age are of particular interest to develop and strengthen strategies for increasing the levels of children’s PA. A qualitative study was conducted to investigate the views of primary school-aged children, their teachers and parents regarding barriers and facilitators to engage in PA.

**Methods:**

Focus groups were conducted separately with primary school children, parents and teachers in a city in Northern Germany between October 2021 and January 2022. The semi- structured focus groups with children and teachers took part in person within school, whereas the focus groups with parents took place online. Data were transcribed verbatim and analysed using thematic analysis. During analysis, the socio-ecological model was identified as useful to map the determinants mentioned and was consequently applied to organize the data.

**Results:**

Teachers (*n* = 10), parents (*n* = 18) and children (*n* = 46) of five primary schools in Germany participated in the focus groups. Participants of the three groups identified similar barriers and facilitators of PA in primary school-aged children, ranging across all four layers of the socio-ecological model. The barriers encountered were the preferences of children for sedentary activities (individual characteristics), the preference of parents to control their child’s actions (microsystem), a lack of financial resources from parents and long sitting times in class (mesosystem), and barriers related to rainy weather and Covid-19 restrictions (exosystem). Facilitators mentioned were the childrens’ natural tendency to be active (individual characteristics), involvement and co-participation of parents or peers in engaging in PA, support provided by teachers and the school (microsystem), living in rural areas, having sufficient facilities and favorable weather conditions (exosystem).

**Conclusion:**

A range of determinants promoting and hindering PA, ranging across all layers of the socio-ecological model were identified by children, parents and teachers in this study. These determinants need to be kept in mind when developing effective PA intervention programs for primary school-aged children. Future interventions should go beyond individual characteristics to also acknowledge the influence of childrens’ social surrounding, including parents, peers and teachers, and the wider (school) environment.

## 1 Introduction

According to the World Health Organisation (WHO), physical inactivity is the fourth leading population-based risk factor for global mortality (1). Regular physical activity (PA) during childhood has been shown to have multiple benefits for childrens’ health, such as for improving their cardiorespiratory and cardiometabolic health and reducing their risk of adiposity and symptoms of depression (2). The WHO recommends children and adolescents to achieve at least 60 min of moderate- to vigorous-intensity of PA per day (3). Results of the German Health Interview and Examination Survey for Children and Adolescents 2014–2017 (KiGGS Wave 2) showed that only 22.4% of girls and 29.4% of boys aged three to seventeen meet the levels recommended by the WHO (4). These rates underline the importance of promoting PA among children in Germany.

Previous studies suggest that PA behaviours track from childhood to adolescence and adulthood (5). Therefore, it is important to promote PA at a young age. Data collected from children themselves is needed to understand the determinants of PA behaviours among children. In addition, involving teachers is important because schools have been identified as an important setting to promote PA (6) since children aged 6–10 years spend a considerable amount of time at school and potentially a large number of children can be reached through schools. Nonetheless, the home environment can also be considered as an important place for children to develop health behaviours (7). Consequently, the parents’ perspective also plays an important role in the promotion of PA in children.

Several qualitative studies with parents, teachers or children on barriers and facilitators of PA have already been published (8–17). Previous qualitative studies investigated parent role modeling, parental support (8–11, 14–18), childrens’ preference for being active (8–10, 15, 16, 19), organised activities and living in rural settings (8–10, 12, 13, 15–17, 20–22) as facilitators of PA in children. Important determinants that hinder PA identified in previous studies have been adverse weather conditions (8–13, 15, 16, 22, 23), costs associated with participating in PA (8, 9, 14, 16), lack of equipment (8, 12, 16), safety constraints (8–10, 13) and lack of parental time to help their children being active (8–10, 15, 16). Based on a focus group study with parents in Spain, children’s PA is influenced not only by barriers and facilitators related to individual determinants, but also to contextual determinants related to friends, parents, siblings, schools and the children’s environment. Unfortunatley, the perspectives of teachers and the children themselves were not included in the study (8). However, even though previous studies have already gathered knowledge on determinants promoting and hindering PA in children (10–13), to our knowledge, no focus group has explored the facilitators and barriers of PA for children in primary school by including the perspectives of teachers, parents and primary school children themselves in Germany concomitantly within one study. The aim of our study was to identify factors that promote and hinder children’s PA by triangulating the views of primary school-aged children, their teachers and parents. In summary, our study allows for a direct comparison of the views of children, teachers and parents based on the same PA intervention, potentially disclosing unknown barriers and facilitators compared to previous studies.

## 2 Methods

### 2.1 Design

A qualitative study using focus groups was conducted with children, their parents and teachers to explore barriers and facilitators of PA in primary school children. This qualitative study was part of a larger German PA research project funded by the Ministry of Health in Germany (BMG): The ACTIvity PROmotion via Schools (ACTIPROS) project, aiming to promote PA in school children aged 6–10 years old by implementing a toolbox of evidence-based PA interventions (see also www.actipros.de). In a feasibility study, we applied and tested the toolbox approach with 12 evidence-based interventions to promote physical activity in children. The interventions included in the ACTIPROS toolbox were activities such as active breaks during and between school hours, physical education or active travel to school initiatives as well as interventions that include a combination of different components in the sense of a “whole school” approach (24).

### 2.2 Sample selection, recruitment, and ethics

This qualitative study is part of a pilot study involving 10 schools (5 intervention schools, 5 control schools) which was conducted to investigate the feasibility and acceptability of using the ACTIPROS toolbox approach for PA promotion (24). In Bremen, the education authority categorizes the school system using an annual index related to social indicators. The ACTIPROS intervention and control classes were matched by the area-level deprivation index to cover Bremen in all five ranks, ranging from 1 – highest social index, to 5 – lowest social index. In this pilot study, two classes at each intervention site were asked to implement the ACTIPROS toolbox over the course of one school year (Nov 2021 until July 2022). Classroom teachers of the 10 intervention classes involved in the pilot study were invited via email to participate in the focus groups. As a focus group of up to 10 participants provides sufficient speaking time for each and at the same time allows a certain group dynamic (25), a selection of 10 children maximum per school were invited to each children focus group by their classroom teachers. Additionally, all parents of participating children from the intervention group (*n* = 217) were further invited to take part in online focus group discussions. Further information on the recruitment and participants of the ACTIPROS study can be found elsewhere (24).

Prior to each focus group, information on the aim of the study and the approximate duration of the focus group were provided and all participants were informed that they could withdraw at any time without negative consequences. Pseudonyms were used throughout the report to preserve the anonymity of participants.

Classroom teachers of the intervention schools taking part in the ACTIPROS study were involved in the recruitment of children and parents. All classroom teachers were invited to a virtual meeting in which information about the purpose of the focus groups and process was provided. Instructions were given in that, e.g., the aim of the focus groups was to gain insight into diverse and different views on PA and that the selection of students and parents should try to contain a heterogenous sample. Five students aged 6–10 years and, separately, all parents of each class were invited to participate.

Ethical approval for this study was granted by Ethics Committee of the University of Bremen, Germany (reference: 2021–17). Parents of all participating students provided written informed consent for their children to take part in the research study. Oral consent was sought and recorded from parents and teachers who took part in the study.

### 2.3 Focus groups

The focus groups took place between October 2021 and January 2022. Separate focus groups were organized for teachers, parents and children to ensure that all groups could talk freely about their perspectives on determinants promoting and hindering students’ PA. Between two and eight participants took part in each group to include a diversity of opinions and perspectives, and to facilitate optimal interaction between participants. All focus groups were conducted by one researcher (LS) with an assistant member of the research team present to take notes. Three topic guides with similar questions were used to direct the focus groups, appropriate to children and adults. The questions directed to children were related to the following issues: enjoyable activities during school and leisure time, active transport, PA opportunities and equipment and suggestions for improvement. The content of focus groups with teachers focused on PA teacher trainings, movement-related activities and excursions, determinants which may be important for the implementation of PA in primary schools and recommendations for sustainable promotion of PA. Parents’ questions centred on children’s movement offerings, family PA, parental participation and determent promoting and hindering their children’s PA.

Focus groups with the children as well as classroom teachers from each of the school classes participating in the ACTIPROS study were conducted in person in a quiet room at the respective schools. The focus groups took place during the school day and a time was arranged in cooperation with the classroom teachers. The duration of the focus groups with children ranged from 29 to 50 min and the focus groups with teachers lasted 12–25 min.

Focus groups with parents of the participating students were conducted via virtual meetings using Zoom at a time and date arranged with parents’. The duration of the parent focus groups ranged from 26 to 40 min.

### 2.4 Data analysis

Focus groups were recorded with an encrypted audio recorder and transcribed verbatim. The available transcripts were initially cross-checked with the original recordings by a member of the research team (LS) and then anonymised. Afterwards, the anonymised transcripts were imported into MAXQDA (Version 20.4.1) to help organize and manage the data and facilitate data coding. The data were analysed using a combined technique of inductive and deductive thematic analysis for identifying themes. The transcripts were coded and first considered separately to get an overview of the perspectives of each participant group (i.e., children, parents and teachers). Similar codes were then grouped together into key themes. Two researchers (LS, HB) independently read a selection of the transcripts and met regularly to discuss the meaning of the codes and key themes generated.

Within the present study, triangulation occurred based on the data source as different groups of participant groups were involved. Whilst the overall methodology of data collection stayed the same throughout semi-structured focus groups, the format of data collection differed to best reach each participant group.

Data collection and analyses occured concurrently. Initially, the coding for each participant group was done seperately, and then all codes were compared and contrasted and integrated into one overall coding scheme. The coding scheme was then applied to all transcripts and it was noted which themes were identified by which participant group.

In the analysis stage, the socio-ecological model was identified as a suitable and fitting model to represent and organize the key themes identified (26). The social-ecological model provides a comprehensive framework for analysing multiple personal and environmental determinants influencing health behaviours (27). It includes 4 levels of environmental influence: (1) individual, (2) microsystem, (3) mesosystem and (4) exosystem (26). As such, all identified determinants were grouped to one of the four levels as per the socioecological model: individual characteristics (5 themes), microsystem (7 themes), mesosystem (6 themes) and exosystem (2 themes). Focus groups were conducted in German; all quotes used in this study were translated in English and were checked by two researchers (LS, HB) and then translated back into German to verify the accuracy of translation. Participant quotes are presented in parentheses to illustrate the respective themes. Quotations provided within this text are marked according to the focus group number (FG = focus group) and type of participant (C = Child; T = Teacher; P=Parent) (see Supplementary file 1).

## 3 Results

A total of 12 focus groups with 74 participants were conducted. Forty-six children participated in five focus groups, up to 10 children per focus group. Another five focus groups were conducted with 10 classroom teachers. Eighteen mothers of primary school children participated in two focus groups. Teachers of three schools located in a low socioeconomic status area, as indicated in the local deprivation index, stated that their students’ parents were not interested in participating in a focus group and did not provide a reason for this.

**Figure 1:**
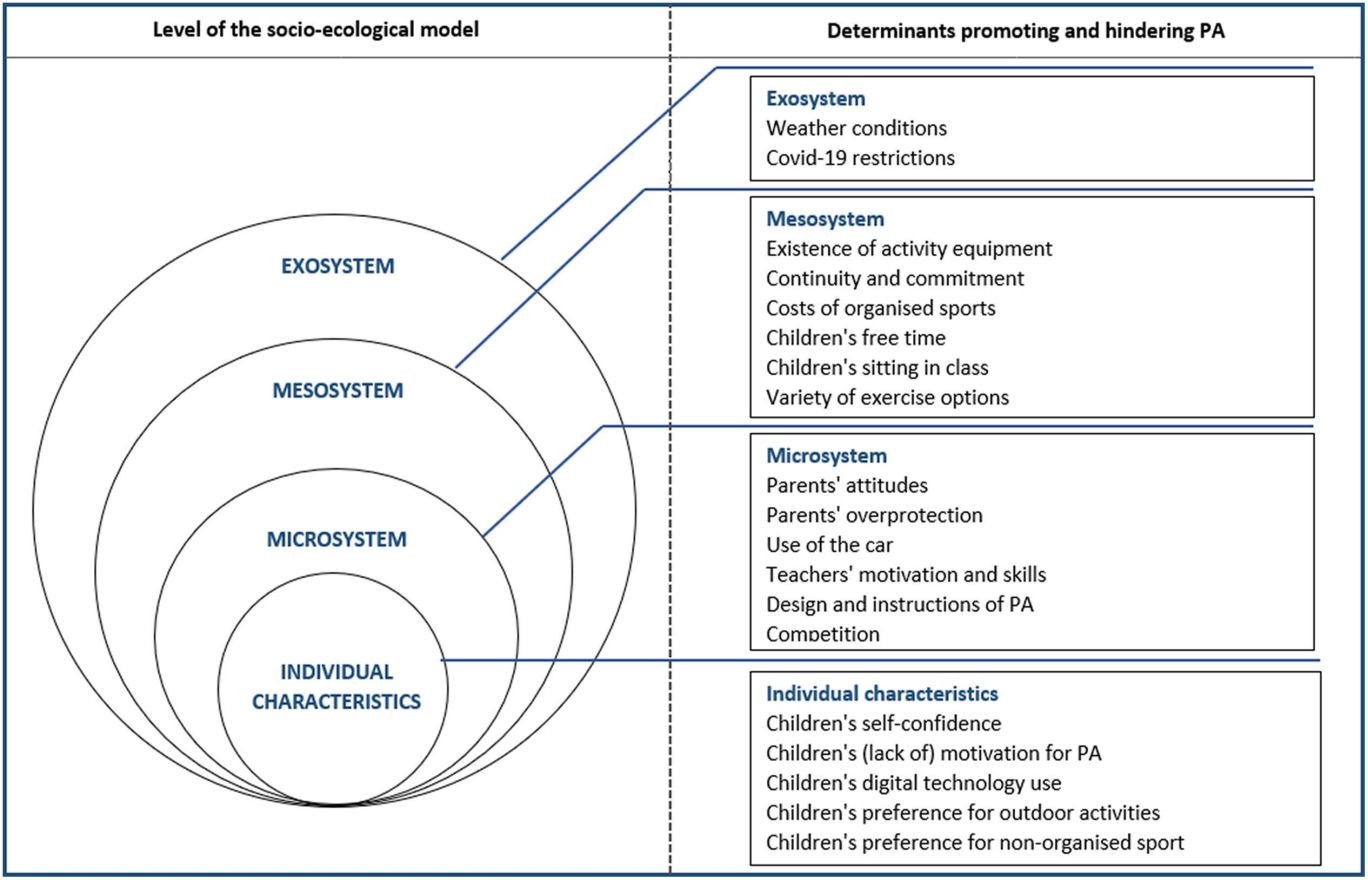
Determinants promoting and hindering PA based on focus groups consisting of children, teachers and parents by level of the socio-ecological model.

The findings of this qualitative study were organized using the following levels of the socio-ecological model: individual characteristics, microsystem, mesosystem and exosystem (Figure 1). The socio-ecological framework outlines four levels of interconnected layers within an ecology. The levels start with the individual and move in concentric circles outwards through the microsystem, mesosystem and exosystem. Bronfenbrenner’s framework (27), with its four layers, serves to recognize that in any school setting, children and their PA are part of a larger whole that is influenced by formal and informal groupings and overarching systems. Nineteen overall determinants stated by teachers, parents and children were identified as barriers and/ or facilitators of PA (see Supplementary file 2).

### 3.1 Individual characteristics

Starting with the inner layer of the socio-ecological model, focussing on individual characteristics, five key determinants were referred to in the focus groups: children’s level of self-confidence, children’s (lack of) motivation for PA, children’s digital technology use, children’s preference for outdoor activities and children’s preference for non-organized sport.

#### 3.1.1 Children’s self-confidence

According to some teachers, self-confidence is a facilitator of PA among children. While activities focused on children’s strengths were seen to encourage children’s engagement in activity, activities that exceed the children’s competences were seen to have a demotivating effect on the children.

*“Anything that encourages children to show their strengths is definitely advantageous.”* (FG5, T1).

#### 3.1.2 Children’s (lack of) motivation for PA

Most of the participants, both parents and teachers as well as the children themselves, stated that children have a natural inner urge to be physically active, they generally feel the need to move.

*“They always want to keep moving, and even if they should already set up, half of them still keep playing because they want to be actively running around all the time.”* (FG3, T1).

However, participants also stated that the motivation to be physically active varied among the children. According to many participating teachers, some children were not as motivated as other to be active, which was particularly evident in their lack of participation in physical education (PE) classes. Some children noted that sometimes they prefered sitting, as during lessons or on their way to school by car. Some parents also reported that their children did not express the need for activities of their own accord during leisure time or that they just prefer sedentary activities rather than being physically active.

*“During class, it’s also great when we sit.”* (FG6, C1).

*“My daughter goes to ballet once a week for an hour, and that’s about it in terms of sports. She’s not the most active child.”* (FG11, P4).

When describing children that were not as active, this was put down to them “not feeling like it” or not “wanting it” by teachers.

*“I think sports courses are chosen by those who have the desire for them anyway. So there are children here at school who go back to the sports club after school or take up other sports offers. And there are also children who do not because they do not have the desire for it.”* (FG1, T2).

A few parents, teachers and children reported that badges in any form, e.g., stickers, certificates, checklists, medals, were seen to have a motivating effect on the children: *“Licenses, certificates and things like that are always very motivating at the elementary school age.”* (FG1, T2).

#### 3.1.3 Children’s digital technology use

Some participants marked children’s preference for digital technology use in their leisure time as a barrier to being physically active. Although one child mentioned exercising via a tablet, when asked what would hinder their movement, the children named a number of digital technologies as barriers to PA, for example mobile phones, tablets, televisions, video game consoles. A parental monitoring of children’s digital technology consumption was regarded by children to possibly facilitate children’s PA level: “*One can tell the parents: `Mom, hide my mobile phone.’*” (FG7, C11). A few parents equally referred to discussions with their children and being active rather than spending time on digital technologies. In addition, from a teacher’s point of view, the low participation of children in sports clubs is seen to be related to increased media use of children.

*“I have got this app on my mother’s iPad: one can do sports with it.”* (FG8, C3).

*“Sitting in front of end devices for hours at a time […] is not exactly conducive to fitness.”* (FG2, T2).

*“And since this [PA] is a competitor to media, I’m a little critical of that.”* (FG11, P4).

#### 3.1.4 Children’s preference for outdoor activities

Almost all children showed a clear preference for outdoor activities compared to indoor activities, both in school and during leisure time. Most parents shared the view that the children prefer outdoor activities.

*“How you could move even more would be to go outside a lot and do a lot of gymnastics and play.”* (FG 9, C1).

#### 3.1.5 Children’s preference for non-organized sports

Both within the school setting and during free time, the majority of children enjoy having free play time and prefer playing without adult guidelines. Some parents remarked that their children barely have any leisure time and opportunities for spontaneous leisure activities.

“*They are allowed to play completely free at the beginning of physical education. They like that.”* (FG3, T1).

### 3.2 Microsystem

Regarding the second layer of the socio-ecological model, representing the level of relationships and social interactions that a child has including his/her family, peers and teachers, the following six key themes were identified: parents’ attitudes toward PA and related behaviours, use of the car, teachers’ motivation and skills, design and instructions of PA and competition.

#### 3.2.1 Parents’ attitudes

Some teachers marked the children’s home as an important environment for PA and reported various opportunities of parental involvement in PA (e.g., PA homework, school garden projects, fundraising runs).

While almost all participating parents overwhelmingly attached great importance to exercise and stated that they wanted to support their children in the best possible way in pursuing physical activities, they suspected that this may not be the case in all families: *““Now we are probably all parents who attach importance to this and look at what the children are doing. And others may possibly not care and say, ‘Fine, then they’ll just goof around the whole day.’. And then do not attach importance to the fact that there are certainly also games where one would have to move around.”* (FG 12, P1).

However, according to some teachers, many parents were seen to not participate in activities such as school events, and teachers perceive this as an important barrier for children’s uptake of PA. Possible reasons cited by teachers for this include parents’ unwillingness, anxiety and convenience to accompany their children to organized activities and participate in activities. Furthermore, some teachers and all parents noted that while many parents were engaged, their work and other commitments did not allow them to participate in PA events themselves or to support their children in PA by accompanying them or picking them up.

*“Sometimes one just do not feel like exercising, and then one just simply lays on the couch in front of the TV the whole Sunday. If one has not watched anything all week or something, then we do not do anything.”* (FG 11, P2).

*“[…] basically, to get the children to move, also besides the school, then, I think, it is with the parents. The parental work, it just does not work very well. I think that’s the biggest obstacle.”* (FG 2, T2).

#### 3.2.2 Parents’ overprotection

According to most children and teachers, many children were not allowed to walk to school without adult supervision. Teachers added that various activities, such as active transport to school and children’s participation in club sports, were limited due to the lack of safety perceived by parents.

*“They [parents] only think about their own child’s safety, not that of the general public. ‘I take my child to school safely by car. I do not see if I’m endangering others or not.`”* (FG3, T2).

*“My mother does not allow me to walk [to school], I have yet to turn eight.”* (FG9, C5).

#### 3.2.3 Use of the car

Teachers reported that many parents take their child to school by car and referred to this as a two-fold problem: more cars on the school ground, which limits the safety of students walking to school. According to participating children, a lack of time, long distance to school and rainy weather often led to the use of car on the way to school.

*“I take the car, (…) because it’s faster, but sometimes I have to go there without the car*.” (FG7, C9).

*“I take the car. Because my house is so far away [from school].”* (FG10, C2).

#### 3.2.4 Teachers’ motivation and skills

Participating teachers viewed the support and engagement of teachers as an important facilitator for the promotion of children’s PA.

In contrast, teachers with a low level of involvement in PA promotion could reduce the opportunities for the children to be physically active. Furthermore, a lack of skills and practice in educating PA may lead to less PA tasks provided by teachers.

*“Of course, there are preferences among teachers and staff as well; they are not all as athletically predisposed as us two. There is probably less movement in the classroom.”* (FG2, T2).

*“I cannot really teach sports at all.”* (FG3, T2).

#### 3.2.5 Design and instructions of PA

As a determinant conducive to children’s participation in PA, teachers further reflected upon the importance of an appealing design of activities and the element of choice. Children were seen to be fascinated by movement stories, music-accompanied activities and movement landscapes, such as multi-variant station training. Parents and children named numerous different types of exercise that the children did regularly or as club sports.

*“I think when you have offerings that are particularly engaging, particularly when one kind of sets something up, movement landscapes, equipment, large equipment is always a huge fascination for the children.”* (FG 4, T1).

Some children spoke positively about their participation in the design of PE classes (e.g., choosing activities/ games as a reward). Teachers also reflected that greater participation of children in the choice of PA activities would have a positive effect on levels of PA. The advantage of providing choices to children was also mentioned by a few parents, who explained how they let their children choose different activities in leisure time.

*“And if it was a child’s birthday, they often get to pick what we play that day. Also choosing something is great fun.”* (FG10, C3).

#### 3.2.6 Competition

Opinions varied on whether competition was considered as a facilitator or barrier in improving children’s PA behavior. According to many teachers, children enjoy taking part in sports activities in a group and the thrill of winning in competitive activities. However, from a parental point of view, competitive sports were also perceived as a barrier, since the focus is on the child’s talent instead of trying out different sports.

*“So you now participate three times, and then you are told whether you have talent there or not,’ and I always feel like that it shifts into this competitive mode really quickly.”* (FG12, P1).

#### 3.2.7 Influence of peers

Almost all children and teachers emphasized interactions in peers as a facilitator for children’s PA. Many children reported going to school together as a group, with siblings or friends.

*“Fun and games, they [children] meet with friends outside and get their exercise by wanting to play with others*.” (FG4, T1).

*“I have a running group. It’s three guys from our class. And I always run up the hill with them.”* (FG 10, C3).

### 3.3 Mesosystem

At level of the mesosystem exploring settings, such as schools and neighborhoods, the following six key aspects were identified: Existence of activity equipment, continuity and commitment, costs of organized sports, children’s free time, children’s sitting in class and variety of exercise options.

#### 3.3.1 Existence of activity equipment

Most of the parents stated that no further equipment is necessarily needed for children to be active in nature: “*They can be super active outside, even without a single piece of playground equipment*.” (FG 11, P2). However, playing equipment were perceived to facilitate children’s activity levels. A lack of resources within the school environment was perceived to inhibit children’s PA opportunities.

#### 3.3.2 Continuity and commitment

According to many participating teachers and parents, children require continuity and commitment regarding physical activities. Regular and fixed exercise times may be important, and exercise should be integrated into the everyday life of the children, both in school and leisure time.

*“I think it’s important that you keep trying, that you really pull it off continuously so that it’s totally normal for the children. And not somehow like `Oh God I have to change again now’, but no, it’s everyday life.”* (FG1, T1).

#### 3.3.3 Costs of organized sports

A few teachers and parents shared the view that a lack of financial resources may also hinder children’s participation in sports. By providing low-cost or free programmes that do not require any additional material, it can be ensured that all children have the opportunity to participate and try out different sports for themselves.

*“The parents do not see the opportunity or cannot do it financially. Because it is always a cost factor if someone plays sports.”* (FG12, P2).

*“There are children who go to the sports club after school or participate in other sport offerings. And there are also children who do not do that because of money*.” (FG 1, T2).

#### 3.3.4 Children’s sitting in class

According to a great number of children, sitting times in class are too long. A large proportion of the children explained that they were generally not allowed to move around in the classroom. From the teachers’ perspective, pure sitting times can be reduced by offering lessons in which the children can alternate between sitting, lying down and walking.

*“In class, we always sit*.” (FG 8, C10).

*“I work with the ‘flexible seating’ system in the classroom. They can choose their workstation and see if they want to work whilst sitting, lying down, standing up, or what suits them best.” (FG1, T2).*

*“…some children actually start moving around in their seats, we have these swivel chairs, they then start spinning around or some start lounging under the table, so they then look for possibilities to move around.”* (FG1, T1).

#### 3.3.5 Variety of exercise options

According to the majority of parents and teachers, providing a wide range of exercise opportunities at school, in after-lunch care, in sport clubs and as a vacation activity can be beneficial for children’s PA. Most parents stated that organized exercise opportunities could be more differentiated in order to encourage their children to be physically active. A few parents remarked that for some specific sports that there were long waiting list (e.g., for swimming): “*I just tried again, for example, to get a place in this [name of course], where children can try out different sports, but I was told that the waiting lists are probably at least two years long.*” (FG 11, P3). Furthermore, some parents critically spoke about the after-school PA offers that they felt were not chosen appropriately because of less variety of content and time overlap with school lessons. Parents expressed the desire for various vacation camps and after-school activities organized by schools or activities that are timed to coincide with school hours, as well as taster courses offered by associations. Therefore, a broad spectrum of opportunities offered in terms of time and content of PA opportunities were seen to facilitate children’s participation in PA.

*“[…] it’s helpful to have different offerings. So if I do not like balancing or running or anything else, just show somehow that sport can be very diverse.”* (FG1, T2).

*“We have a very large and well-known sports club here right next to the school. But I actually think that the sport offers for children could be even more differentiated. So my kids do not find anything there [at the sports club] straight away.”* (FG11, P5).

#### 3.3.6 Gardens, sport clubs and parks close to home

Both children as well as a few parents report that their own garden, basement, green spaces, playgrounds and other opportunities for movement in the residential environment are frequently and gladly used by the children for movement. A residential environment with exercise areas/green spaces provides opportunities for exercise. In addition, the children can walk unaccompanied. More distant opportunities for exercise are often an obstacle for parents, because they are connected with journeys. The residential environment also determines whether children actively make the journey to school.

*“I think we are also very privileged, simply in terms of the living environment. So the kids, I think, all have the opportunity to get out relatively quickly, to get somewhere in nature. So we all have a hiking trail across the street more or less, so that’s all relative that you can say, `There’s an opportunity there, too.’ So that is not reliant on driving there.”* (FG 12, P1).

### 3.4 Exosystem

Two key aspects were referred to the level of the exosystem focusing on structures: weather-conditions and COVID-19 restrictions.

#### 3.4.1 Weather conditions

When speaking about barriers to PA, many discussions also referred to the (bad) weather conditions. Bad weather was particularly often used as a reason to not engage in active travel to school. Most children and parents said they spend more time active outdoor when the weather is good like in summer and spring and more time being sedentary when it rains.

*“I always come by bike, but if it’s raining or something, my mom takes me to school by car.”* (FG 7, C11).

#### 3.4.2 COVID-19 restrictions

Several teachers, parents and children reported negative effects of Covid-19 restrictions on children’s PA, such as sports classes not taking place and PA offerings at school being cut or canceled due to contact restrictions.

*“If I now had Corona, I would not be able to go outside. And then, I do want to move around a lot, but if I had Corona now, I could not.”* (FG 10, C1).

Looking across all levels of the socio-ecological framework, children, teachers and parents identified similar barriers and facilitators to PA for primary school children. Most determinants addressed pertained to the levels “Individual characteristics,” “macrosystem,” “mesosystem” and only two determinants were identified that related to the “exosystem.”

## 4 Discussion

The aim of this study was to explore the individual, social and organizational determinants influencing children’s activity behaviors, from the perspectives of children, parents and teachers. The results of this study show that according to all three groups PA of children aged six to 10 years is influenced by all levels of the socio-ecological model, namely individual characteristics, microsystem, mesosystem and exosystem. The promotion of PA may be facilitated by actions at a variety of levels across multiple domains, as both promoting and hindering determinants were mentioned at all levels. Below, the frequently reported determinants are discussed in relation to previous studies followed by implications for future research and potential interventions.

In relation to the individual characteristics and in line with previous studies (8, 9, 16, 19, 28), teachers, parents as well as children themselves considered children as very active and full of energy. However, this contradicts the findings that many children do not achieve the recommended minimum amount of daily PA (4). Although all subgroups perceived digital technology use as a determinant hindering children’s PA, one participating child noted that digital technologies can improve children’s PA by using an app to participate a digital sports programme. Oh et al. concluded in their review of digital interventions that there is great potential in digital platforms for health promotion in children (29). One study also showed that digital media may play a two-sided role when it comes to PA, enabling the promotion as well as presenting a barrier to PA (30). Although children and parents reported that outdoor activities are preferred by children, children themselves describe parents and teachers as not always allowing them to play outside, e.g., because of weather conditions or safety constraints. Since contact with green spaces has been shown to have positive effects on PA, the promotion of outdoor PA irrespective of certain weather conditions within the school setting as well as during leisure time, might lead to increased levels of children’s PA (31).

When comparing the perspectives of parents, teachers and children in relation to individual determinants, it becomes clear that children have a natural motivation to be physically active. In all focus groups, children’s preference for non-organized physical activities was mentioned. While all three groups noted hindering aspects of the use of digital technologies, a benefit was also mentioned from the children’s perspective. The promoting aspect of outdoor activities was mentioned by parents and children but not by teachers. They, however, were the only ones to state the importance of children’s self-confidence regarding their PA.

About the microsystem, and in line with previous studies (8, 32), the results of this study show that support, involvement and encouragement of parents, teachers, siblings and peers can have positive effects on childrens’ PA levels. If parents have a positive attitude in supporting PA, children tend to become more physically active (21). However, parents prefering to control their child’s actions were perceived as a barrier to PA by children and teachers. Suen et al. reported that safety concerns discouraged PA of young children (33). Therefore, future research should better explore the influence of parents’ overprotection and its influence on childrens’ habitual PA. In line with other studies, parents’ use of the car was identified as hindering PA by children and teachers. Due to the short distance between the school and the home of the participating children in this study, it could be concluded that the perception of distances is a very subjective matter. Several studies have demonstrated that individual factors such as a child’s age are crucial in relation to children’s independent commuting (34, 35). The switch from parent-accompanied to independent commuting may be an important entry point for PA promotion of children that has been underutilized in Germany so far. A childlike and playful approach was perceived to be facilitating the PA of children. Activities including competitions were perceived as both, a hindrance and a benefit. Despite this, teachers and children perceived sitting times in class as a barrier of being physically active. Further studies confirm these findings by showing that children sitting time during school hours is longer compared with sitting times in non-school hours (36, 37).

All three groups perceived that children’s closest social circle-peers and family members influence their PA behavior. Teachers also emphasized the promoting influence of teachers’ motivation and skills. Only children and teachers mentioned parents’ overprotection and use of the car as hindering determinants. All groups agreed on the promoting influence of a playful, child-friendly design of PA. Parents and teachers mentioned both facilitators and barriers regarding competitions in sports activities.

Regarding the mesosystem, a key facilitator was to provide children with various PA options. Variety included school-based and after-school programs, individual or team sports, competitive or non-competitive activities and exercise opportunities at different times. According to the parents and children, gardens, sport clubs and parks close to children’s home facilitated PA. These results are consistent with a study (8) that showed household and neighborhood factors encouraged PA in children.

While all groups perceived the availability of play equipment and free playtime as promoting determinants for children’s PA, teachers and parents also noted the influence of varied options of exercises as well as the cost and continuity of sports.

In relation to the exosystem, the results of this study are congruent with previous studies that confirm that bad weather conditions limit the level of PA of children and their time for outdoor play (8, 12, 16, 22, 23). In this respect, most of the children reported that they were not allowed to play outside during bad weather seasons. In line with other studies (8, 16, 18, 22, 23, 38, 39), our results reflect that bad weather conditions encourage the use of the car for transportation to school, even in urban areas where distances are short. A possible explanation for the low participation in active traveling to school in this study could be the urban conditions, such as large intersections or few opportunities to cross the road. The present study was conducted with children living in an urban area in Germany, where factors influencing active travel might differ from rural populations. Future research is needed to explore strategies to reduce the use of cars in bad weather. At the same time, it is important to explore whether or to what extent active transport is feasible and desirable for all children. As our results have shown, other approaches to increasing PA are desirable, such as providing a variety of physical activity options. Moreover, participants of all three subgroups perceived Covid-19 restrictions as a barrier to PA, such as needing to stay in quarantine. These findings are in line with a study by Kovacs and colleagues investigating the impact of the COVID-19 pandemic on PA in European children and adolescents aged 6–18 years old. Kovacs et al. suggested that children’s PA level decreased dramatically during winter lockdown (40).

Regarding the exosystem, the Covid-19 restrictions were perceived by all three groups as a barrier of children’s PA behavior. Children and parents also mentioned bad weather conditions as a hindering factor.

The results of the present study provide an in-depth view and several implications for research and practice from parents, teachers and children on determinants facilitating and hindering PA in children aged 6–10 years old. While teachers, parents and children identified the same facilitators and barriers, there were differences in their perceptions of these perceived determinants promoting and hindering PA in children. These findings suggest that there is a need to include different perspectives in future research when designing PA interventions. According to the results of this study, parental encouragement and support were perceived as facilitators of children’s PA. Thus, including parents in the development and implementation of physical activities is needed. Moreover, peers and siblings should be involved in promoting children’s PA as they seem to be a facilitating factor for children’s participation in physical activities. Since children prefer outdoor activities, they should be offered the opportunity to be active outdoors regardless of any weather conditions. By introducing policies at schools that aim at ensuring that children can exercise outdoors during recess independent of the weather and offering them sufficient time for PA without instruction, PA could be improved in school-aged children. The design of active transport to school cannot be based on a “one size fits all” approach, but contextual determinants such as the children’s living environment, the parents’ need for safety, dealing with weather conditions, and scheduling should be considered. Traffic density often occurs around schools in Germany because parents drive their children to school for safety reasons (41). In order to promote active travel to school, the safety risks assumed by parents should therefore be reduced. Besides, most accidents on the way to school in Germany occur in cars being driven to school and not walking or cycling (42). Policy makers and traffic planners are still needed to promote active transport to school for children.

The strength of our study is that it adds to the available literature by exploring the facilitators and barriers of PA for children aged 6–10 years by including the perspectives of teachers, parents and children themselves. To date, qualitative research exploring this topic has predominantly focused on the perspectives of the teachers or the parents (8, 43), but also on parents and children (44, 45) separately. Confronting these three perspectives helped to triangulate the data and provided deeper understanding.

Several limitations will need to be acknolwedged. A first limitation of the study was the possibility of selection bias, because participants attended the focus group discussions voluntarily. The most motivated parents and teachers might have participated, whose children already might comply with current PA recommendations. In addition, there is the possibility of a selection bias because we limited the number of children to five participants per school class and these children were selected by the teachers with the aim of including heterogeneous perspectives. Furthermore, in the group of parents, focus group participants were entirely female. The experiences of fathers is therefore missing within the analysis. Despite concerted efforts to recruit a diverse range of parents, this was not achieved in the current study. Since, there is evidence relating to the role of the father in a child’s development (46), it would be a valuable addition to the body of research to gain the fathers’ perspective on factors that promote or hinder their children’s physical activity. However, the child samples included both girls and boys of different age groups which provided a variety of perspectives. The focus groups and data analysis were conducted by the same researcher, which increases the risk of bias. However, focus groups were transcribed verbatim and additional researches were involved in both the focus groups and the data analysis.

## 5 Conclusion

There is consistent qualitative evidence that several determinants at various levels of the socio-ecological model influence children’s PA behavior. Our results confirm children prefer non-organized and outdoor activities that involve active movement and play. However, the type of activities undertaken is strongly influenced by the attitudes and motivation of teachers and parents as well as siblings and peers.

PA should be promoted through a combination of intervention components, e.g., by using a socio-ecological framework focusing on the children, their relationships and environment. A comprehensive approach could include supporting PA regardless of actual weather conditions, involving teachers, peers and families, offering various opportunities of organized and non-organized activities in different settings and regulating the use of electronic devices. For the development and implementation of interventions in primary school setting, it is important to take into account the possible hindrances of PA explored in this study. Thus, strategies for regulating children’s digital technology consumption, individual use of competitive situations among children, parental involvement in outdoor activities and active transport of children as well as strategies for physical activity even under pandemic conditions might be necessary.

## Data availability statement

The raw data supporting the conclusions of this article will be made available by the authors, without undue reservation.

## Ethics statement

The studies involving humans were approved by the Bremen University Ethics Committee. The studies were conducted in accordance with the local legislation and institutional requirements. Written informed consent for participation in this study was provided by the participants’ legal guardians/next of kin.

## Author contributions

LS: Writing – original draft, Data curation, Formal analysis, Methodology. MB: Writing – review & editing, Conceptualization, Project administration, Funding acquisition, Data curation. BB: Writing – review & editing, Project administration. HZ: Writing – review & editing, Funding acquisition. HB: Writing – review & editing, Methodology, Supervision.
