# Supplementary material for: Determinants promoting and hindering physical activity in primary school children in Germany: a qualitative study with students, teachers and parents
Source: Front Public Health. 2024 Feb 2;12:1280893. doi: 10.3389/fpubh.2024.1280893 (PMC10869510; doi:10.3389/fpubh.2024.1280893)
Supplement: Supplementary file 1 [file Table_1.DOCX]

**Additional file 1. Quotes from focus group discussions**

| **Verbalizations** | **Category raised by T= Teacher,  P= Parent,  C= Child** | **Description** | **Categories** |
| --- | --- | --- | --- |
| **Individual Determinants** | | | |
| "Self-confidence is one thing that helps in sports. If you just think about yourself and can block out everything else, then it works better and then you also do everything."  (FG 2, T2) | T | Children’s self-doubt may hinder children’s physical activity, while strong self-confidence proves beneficial. | **Children’s self-confidence** |
| "I do have the feeling that they have such an inner urge to move, to romp, to fight, to run, that they actually always want to be in motion. Definitely, I just notice that with my class, that they're actually always, always move around."  (FG1, T1) | T, P, C | A child’s inner urge to be physically active, enjoyment of PA, and any form of badges may increase their PA  level. In contrast, children’s preferences on sedentary activities  may negatively influence children’s participation in PA. | **Children’s (lack of) motivation for PA** |
| "...and the fact that this tablet was also provided by the school, I have to honestly say that sometimes we have trouble with that discussion where it's like, well, are we really going to go out and do something together, or am I still sitting here doing something on the tablet?"  (FG 11, P4) | T, P, C | Using digital technology may hinder children being active. | **Children’s digital technology use** |
| "Playing outside is always more fun for me. Because you can do much, much more in the garden than indoors." (FG 10, C3) | P, C | Children prefer outdoor activities. | **Children’s preference for outdoor activities** |
| "I particularly enjoy the fact that while [name of the teacher] is checking her planner, we have time for ourselves and play freely. And that's a lot of fun for us […]" (FG 10, C3) | T, P, C | Children prefer non-organised sports. | **Children’s preference for non-organised sports** |
| **Microsystem** | | | |
| "I think it would be important, especially in the home environment, that at home, I say, exercise is given a higher priority [...]" (FG 4, T1) | T, P, C | A child’s PA participation may be  facilitated through support and involvement of parents and  family activities. | **Parents´ attitudes** |
| “I often have the feeling that parents tend to be very anxious […] Even when it comes to going to school alone.” (FG 3, T2) | T, C | Parents preference to control their child’s actions and avoiding danger may hinder their PA. | **Parents´ overprotection** |
| "...many children, of course, live here in the immediate surroundings, but are still driven by car to the classroom if possible...". (FG 4, T1) | T, C | A child’s activity may be hindered by the use of car. | **Use of the car** |
| "If oneself is interested, then of course one can get the children to be more involved, that's very clear."  (FG 1, T 2) | T | PA opportunities provided by supportive teachers may facilitate children’s participation in PA. In contrast, perceived  lesser value of PA and a lack of skills in teaching PA may negatively influence the children’s PA. | **Teachers´ motivation and skills** |
| "But somehow I try to be a little bit responsive and choose songs that are age-appropriate. And then that actually goes down well with them, I can motivate them with that."  (FG2, T1)  "So I think what would be great [...] if the children had even more of a say in these topics."  (FG 3, T1) | T, P, C | A childlike and playful approach with various games, music and stories may facilitate children’s participation in PA.  Children’s PA may be facilitated by involving them in PA opportunities. | **Design and instructions of PA** |
| "In sports, one can see particularly well what one is good at, one can compare oneself with other children, and that's what they measure themselves against. All the competitions are actually motivation enough."  (FG2, T2) | T, P | Competitive activities may facilitate children to be active but may also hinder children from trying out different activities. | **Competition** |
| "It's the togetherness that I think is particularly important for the children." (FG2, T2) | T, P, C | Children may be more likely to take part in PA when there is involvement of peers. | **Influence of peers** |
| **Mesosystem** | | | |
| "It's a pretty spacious schoolyard where they also have a lot of opportunities to do things. [...] Because one can also borrow equipment and so, so one has definitely opportunities to exercise during recess."  (FG12, P1) | T, P, C | Play equipment were perceived to facilitate children’s activity levels. A lack of resources within the school environment was perceived to inhibit children’s PA opportunities. | **Existence of activity equipment** |
| "[...] it's important that there's at least some regularity in it with some sport that she feels like doing."  (FG11, P4) | T, P | Regular and fixed activities may facilitate childrens’ PA | **Continuity and commitment** |
| "There are children here at school who go to organized sports after school or take part in other sports activities. And there are also children who don't do it because [...] they can't afford it." (FG1, T2) | T, P | A lack of financial resources may hinder children’s participation in sports. | **Costs of organised sports** |
| "Well, I would also like to move more in class because most of the time we are always sitting [...]." (FG 6, C 3) | T, C | Children perceive sitting times in class as a barrier of being physically active. Flexible teaching methods could reduce children’s sitting times in class. | **Children’s sitting in class** |
| "Some sports clubs already offer vacation care, in a mixed form. I think that goes down very well with the kids, too, because they also offer a variety of activities." (FG 11, P1) | T, P | A wide range of organized exercise opportunities can encourage children’s participation in PA. | **Variety of exercise options** |
| "...We don't have enough free time in the sportshall. We only play for about 15 minutes. [...] I would rather have 30 minutes."  (FG7, C5) | T, P, C | Children may be more active when they have spare  time for playing. | **Children’s free time** |
| **Exosystem** | | | |
| "We also often go on bike rides together in the summer; we're fair-weather cyclists." (FG 11 ,P4) | P, C | Weather conditions are influencing a child’s PA. While sunny weather facilitates children’s PA, rain and snow seem to hinder their PA behaviour. | **Weather conditions** |
| “…we just can't simply mix the age groups. A lot has happened because of Covid, and you can just tell with the children - that the movement is just missing.” (FG 2, T2) | T, P, C | Lesser PA opportunities due to covid-19-restrictions may inhibit children’s participation  in PA. | **Covid-19 restrictions** |

**Abbreviations: P = Parents; C = Children; T = Teacher; FG = Focus Group.**
