# Supplementary material for: Determinants promoting and hindering physical activity in primary school children in Germany: a qualitative study with students, teachers and parents
Source: Front Public Health. 2024 Feb 2;12:1280893. doi: 10.3389/fpubh.2024.1280893 (PMC10869510; doi:10.3389/fpubh.2024.1280893)
Supplement: Supplementary file 2 [file Table_2.DOCX]

**Additional File 2. Determinants of children's physical activity identified by children, teachers and parents.**

| **Determinants promoting and hindering children's PA** | **Categories reported by children, teachers and parents** | | |
| --- | --- | --- | --- |
|  | Children | Teachers | Parents |
| Children’s self-confidence |  | x |  |
| Children’s (lack of) motivation for PA | x | x | x |
| Children’s digital technology use | x | x | x |
| Children’s preference for outdoor activities | x |  | x |
| Children’s preference for non-organised sports | x | x | x |
| Parents´ attitudes | x | x | x |
| Parents´ overprotection | x | x |  |
| Use of the car | x | x |  |
| Teachers´ motivation and skills |  | x |  |
| Design and instructions of PA | x | x | x |
| Competition |  | x | x |
| Influence of peers | x | x | x |
| Existence of activity equipment | x | x | x |
| Continuity and commitment |  | x | x |
| Costs of organised sports |  | x | x |
| Children's sitting in class | x | x |  |
| Variety of exercise options |  | x | x |
| Children's free time | x | x | x |
| Weather conditions | x |  | x |
| Covid-19 restrictions | x | x | x |
